# Impact of Printing Orientations on the Trueness and Precision of Additively Fabricated Complete Denture Base Before and After Thermal Aging

**DOI:** 10.3390/dj13120598

**Published:** 2025-12-12

**Authors:** Sara Tarq AL-Zayyat, Turki Alshehri, Shahad T. Alameer, Sarah Hajaj Althunayyan, Reem A. Aldhafiri, Zainab Albasry, Abdulrahman A. Balhaddad, Haidar Alalawi, Mohammed M. Gad

**Affiliations:** 1 Department of Prosthodontics and Implantology, Ministry of Health (MOH), P.O. Box 21217, Riyadh 11176, Saudi Arabia; sara.t.alzayyat@gmail.com; 2 Department of Substitutive Dental Sciences, College of Dentistry, Imam Abdulrahman Bin Faisal University, P.O. Box 1982, Dammam 31441, Saudi Arabia; raaldhafiri@iau.edu.sa (R.A.A.); zahalbasry@iau.edu.sa (Z.A.); haalalawi@iau.edu.sa (H.A.); mmjad@iau.edu.sa (M.M.G.); 3 College of Dentistry, Imam Abdulrahman Bin Faisal University, P.O. Box 1982, Dammam 31441, Saudi Arabia; 2180000445@iau.edu.sa; 4 Department of Prosthodontics and Implantollogy, Ministry of National Guard Health Affairs (MNGHA), P.O. Box 22490, Riyadh 11173, Saudi Arabia; sarahalthinayyan@gmail.com; 5 Department of Restorative Dental Sciences, College of Dentistry, Imam Abdulrahman Bin Faisal University, P.O. Box 1982, Dammam 31441, Saudi Arabia; abalhaddad@iau.edu.sa

**Keywords:** complete denture, accuracy, 3D printing, printing parameters, printing orientation

## Abstract

**Objectives:** This in vitro study assessed the accuracy (trueness and precision) of different 3D-printed resin denture bases with 0°, 45°, and 90° printing orientations. **Methods:** Denture base was designed and fabricated using three 3D-printed denture base resins (DentaBASE, Denture 3D+, and FormLabs). Each resin was printed with its own printer and fabricated with different printing orientations, resulting in a total of 72 specimens (n = 8). Trueness and precision were evaluated before and after thermal aging using the superimposition method with best-fit alignment. The data were collected and analyzed using two-way ANOVA followed by post hoc Tukey’s test (α = 0.05). **Results:** The printing orientation significantly affected the trueness of 3D-printed resins (*p* < 0.001). The highest trueness was observed for NextDent at 0° printing orientation, while the lowest value was observed for ASIGA at 0° and 45° printing orientations. The precision of the denture base was significantly affected by different printing orientations for ASIGA (*p* = 0.006) and NextDent (*p* < 0.001) before thermal cycling, while the precision of FormLabs was significantly affected (*p* = 0.017) after thermal cycling. The highest precision was recorded for FormLabs at 45° printing orientation, while the lowest precision was observed for NextDent at 45° and 90° printing orientations. Moreover, the effect of thermal cycling on trueness was only significant for ASIGA at 0° printing orientation; however, the effect of thermal cycling on precision was significant for NextDent at 0° and 90° printing orientations. A 45° printing orientation provided the most accurate clinical fit. **Conclusions:** ASIGA showed the lowest trueness, while FormLabs exhibited the lowest precision, revealing performance differences between printers.

## 1. Introduction

The use of computer-aided design and computer-aided manufacturing (CAD/CAM) in dental procedures has improved significantly and considerably impacted restorative dentistry [1]. The advantages of removable CAD/CAM prosthesis include significant reductions in required appointments, direct duplication of existing dentures, and enhanced tissue adaptation [2]. Common manufacturing methods for dentures include subtractive manufacturing (SM), such as milling, and 3D printing, such as additive manufacturing (AM) [3]. However, AM is considered to be superior to SM [4], as SM poses several comparative disadvantages, including wear of rotary milling burs, waste of excess material, and challenges in producing complex models [4]. In contrast, the outcomes of AM can be affected by several parameters, such as the printing orientation and angle, layer thickness, and laser speed [5,6].

The AM process consists of three phases: data processing, printing, and post-printing [7]. The process parameters can significantly influence the final properties of the printed specimens [8]. In the pre-printing phase, the type of photopolymerized resin used, its composition, and the photoinitiator are crucial for the 3D printing resin polymerization process [5]. During the setup of the printing phase, several factors can affect the outcome of the printed object, including layer thickness, orientation, support structure, density, position, printing speed, depth of light penetration, and the technology employed [9]. A variety of printing technologies are employed in the fabrication of denture bases. Digital light processing (DLP) and stereolithography (SLA) are the most commonly used printing technologies, impacting the properties and performances of printed objects [10]. Different vat-polymerization techniques result in distinct mechanical and biological behaviors in denture base resins [2]. The employment of diverse technologies, the illumination source of 3D printers, and their printing materials may potentially impact the geometrical accuracy of printed dentures [11]. A comparison of the two printing technologies was previously conducted in two studies by Yoshidome et al. [12] and Unkovskiy et al. [11]. The focus of these studies was on the accuracy of printed denture bases. According to the study by Yoshidome et al., the SLA demonstrates superior fitting accuracy in comparison with the DLP. In contrast, Unkovskiy et al. observed a high degree of precision in both technologies, with SLA demonstrating superior trueness compared to DLP [11,12]. The primary distinction between SLA and DLP lies in the time required to print multiple models concurrently [13]. The simultaneity of the printing of multiple DLPs does not result in an alteration of the time required for printing. However, the most accurate method for printing CDs has yet to be determined. Despite the fabrication of precise dentures by some studies, the influence of support parameters on molding errors remains to be elucidated [14].

To enhance the degree of monomer conversion and reduce residual monomer content, post-printing polymerization cycles are necessary [15]. The post-printing polymerization phase involves multiple variables, including cleaning methods, polymerization time, temperature, curing machines, and the type and wavelength of light used [16].

To achieve optimal clinical outcomes, parameters in the AM workflow must be refined to ensure accurate printed objects; a particularly critical parameter is the printing orientation [17]. Clinical significance has been demonstrated between different printing orientations, resulting in varying outcomes for the same object [18]. In clinical dental procedures, accurately replicating a denture shape created with CAD is essential for ensuring a precise fit in the patient’s mouth [19]. According to International Standards Organization (ISO) standard 5725-1:2023 [20], accuracy encompasses both trueness and precision. Trueness refers to how closely the average of multiple test results aligns with the expected or reference values, indicating the deviation between the dimensions of the newly prepared object and the original printed dimensions. Precision describes the consistency of results across repeated measurements, reflecting the ability to reproduce printed objects with similar dimensions [20,21].

The type of material and the geometry of the denture base significantly affect printing accuracy [22,23,24,25]. Therefore, both printing orientation and component geometry are important design factors [8]. In addition, these variables are not all independent; modifying one variable can lead to changes in others. For example, different printing orientations may require various support structures and positions [26,27]. The interaction between these variables can positively or negatively affect the printing accuracy of the denture base [19]. Various studies have focused on accuracy, material consumption, and processing time of digital manufacturing techniques, also emphasizing the need for high strength [24,28].

Although many studies have investigated the effect of various factors on the accuracy of complete dentures (CDs) using bar specimens [29], which are different from denture configurations, this study evaluated the effects of printing orientations (0°, 45°, and 90°) of different types of 3D-printed resins on the accuracy (trueness and precision) of 3D-printed maxillary denture bases. Previous studies have shown that the performance of 3D-printed objects is influenced by printing orientation. Specimens printed at a 0° orientation contain fewer layer interfaces, which enhances their physical and flexural properties because the applied load acts perpendicular to the printed layers [2]. However, there is limited evidence on how printing orientation and material type collectively influence the clinical accuracy of complete denture bases, particularly after simulated aging. This lack of clarity hinders the optimization of printing parameters for achieving precise and stable dentures. The null hypothesis stated that there would be no differences in accuracy (trueness and precision) among the three different printing orientations and printed resins.

## 2. Materials and Methods

**Figure 1:**
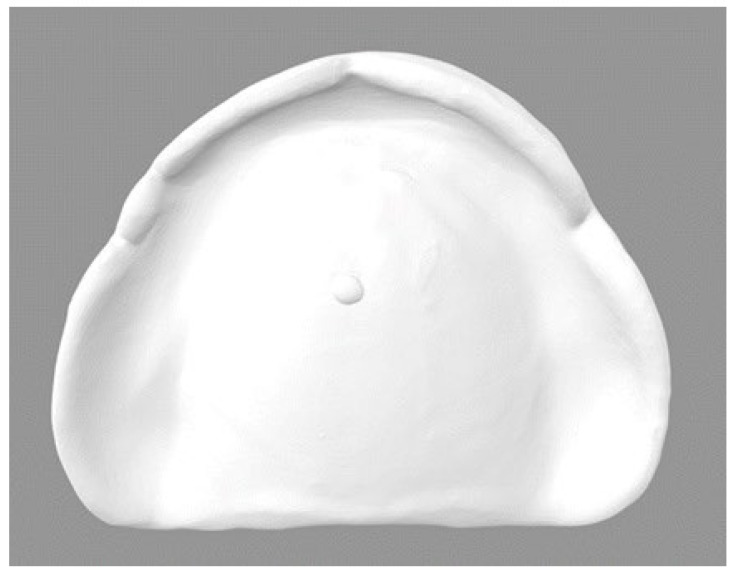
Designed STL file for the scanned denture base.

A power analysis was conducted to determine the appropriate sample size for this study. Based on an anticipated medium effect size, a significance level (α) of 0.05, and a power of 80%, the recommended minimum sample size was calculated to be 8 specimens per subgroup. Accordingly, a total of 72 specimens were included, divided into three main groups according to the type of 3D-printed denture base resin: DentaBASE (ASIGA, Erfurt, Germany), Denture Base Resin LP (FormLabs Inc., Somerville, MA, USA), and Denture 3D+ (NextDent B.V., Soesterberg, The Netherlands). Each resin was further subdivided into three groups (n = 8) based on printing orientation: 0°, 45°, and 90° [2,7]. To create a denture base, an edentulous maxilla model (Nissin Dental Model, EDE1001, Nissin, Kyoto, Japan) was taken using a rubber impression material (Exadenture, GC, Tokyo, Japan). A plaster model was fabricated using type IV plaster (Fujirock EP, GC, Tokyo, Japan), the provided mixing ratio, and a vacuum mixer. The plaster model was then scanned using a 3Shape TRIOS intraoral scanner (3Shape A/S, Copenhagen, Denmark) to obtain standard tessellation language (STL) files representing the digital model of the edentulous maxilla. Utilizing the classification system established by the American College of Prosthodontists, we chose an edentulous upper and lower cast featuring class I, type A residual ridge shapes, devoid of significant undercuts [30]. A CAD virtual design was generated using freely available software (123D design v. 2.2.14; Autodesk, CA, USA). This design was then saved in Standard Tessellation Language (STL) format and transferred to 3D printing software for further processing, as shown in Figure 1.

### 2.1. Specimen Printing

**Figure 2:**
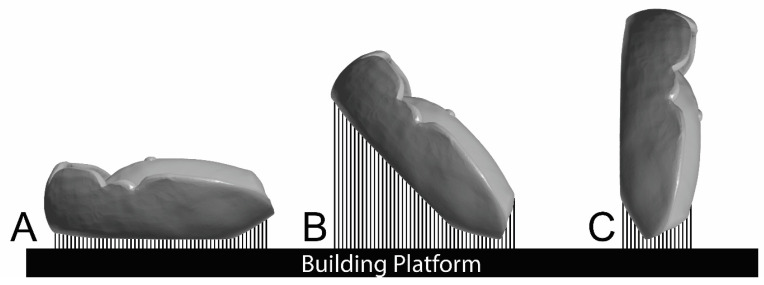
Denture base printing orientations, (**A**) 0°degree, (**B**) 45° degree and (**C**) 90° degree of printing orientations.

Using the STL file, 72 denture base resins were fabricated at 0°, 45°, and 90° orientations via 3D printing; the appropriate 3D printer corresponding to each resin type was employed, as shown in Figure 2. Each denture base was then washed at 20 °C and 50% humidity using 99.9% isopropyl alcohol for 10 min. Subsequently, each group underwent post-curing as per the manufacturer’s instructions, as mentioned in Table 1, considering the appropriate machine, time, and temperature. After curing, the support structures of the denture bases were meticulously removed using a slow-speed rotary instrument (MetaServ™ 250 Grinder–Polisher with Vector™ Power Head (Buehler, Lake Bluff, IL, USA)), followed by standardized finishing with silicon carbide grinding papers (3M™ Hookit™ 260 L, 600–1200 grit, 3M Company, St. Paul, MN, USA). This finishing process was conducted by a single investigator. Before testing, all denture bases were immersed in distilled water for 48 h at 37 °C [29]. The full printing specifications, printers, and parameters are listed in Table 1.

### 2.2. Accuracy Measurement

**Figure 3:**
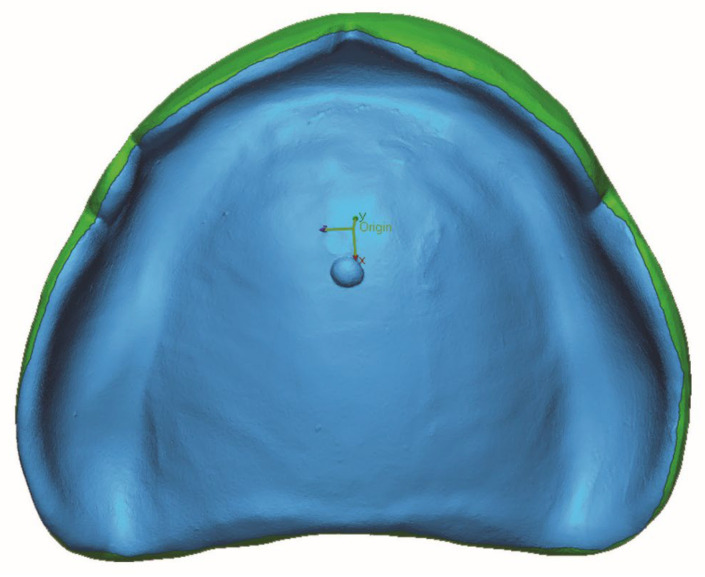
Boundary of the comparison area for accuracy analysis (blue = included comparison area and green = non included area).

The intaglio surfaces of all 3D-printed denture bases underwent scanning using an intraoral scanner (TRIOS 3, 3shape, Copenhagen, Denmark), and the resulting images were saved as STL files. To measure accuracy, each STL file of the denture’s intaglio surface was superimposed with the STL file of the reference model using both initial alignment and subsequent best-fit alignment using a 3D measuring tool (Geomagic Control X, 3D Systems, Rockhill, SC, USA) [31,32]. The comparison was confined to a predefined region of interest delineated from the highest contour of the denture flange; the blue surface in Figure 3 displays the analyzed intaglio surface, with the peripheral (green) flange beyond this boundary excluded from the calculation. Accuracy values were compared by calculating surface deviation values using the root mean square error (RMSE). RMSE was computed as the square root of the mean square, enabling the identification of the error quotient in units like the true value. It provided a measurement of the variability of surface dimensions and shapes.

In this study, RMSE was determined as an error value of trueness, as follows:

RMSE = (1 / √n) · √(∑i₌1ⁿ (x_1,i_ − x_2,i_)^2^)

Here, ×1 and ×2 denote the predicted and actual values, respectively, while 1 indicates the initial term’s order for sum calculation in the formula. Since it begins from the first term, i is fixed at 1. The statistical analysis involved computing the RMSE value using the provided formula. A significant RMSE value, distant from zero, suggests considerable dimensional error compared to the reference model; conversely, a small RMSE value, close to zero, indicates small dimensional error.

### 2.3. Trueness and Precision Method

The scans were superimposed on the STL reference file to assess trueness compared to the original STL reference denture base. Precision was evaluated by collecting data through multiple scans of each printed denture base material, and both were assessed through software and measurement methods (Geomagic Control X, version 2018.0.1; Geomagic Inc., 3D Systems, USA), in which STL files were superimposed on the reference scan [33].

### 2.4. Thermal Aging

Thermal stress was applied to the specimens using a thermocycling machine (Thermocycler THE-1100-SD; Mechatronik GmbH, Feldkirchen-Westerham, Germany). Each specimen was subjected to 10,000 thermal cycles between 5 °C and 55 °C with a dwell time of 30 s each, intended to simulate one year of intraoral use [34]. After the cycling, each sample was remeasured for trueness and precision.

### 2.5. Statistical Analysis

Data normality was checked using the Shapiro–Wilk test, and insignificant results from the tests indicated the data were normally distributed. Hence, parametric tests were used for inferential data analysis. Two-independent samples *t*-test was used to study the thermal cycling (TC) effects on trueness and precision, while one-way ANOVA was utilized to determine the effects of printing orientation and resin type on the tested properties. In addition, the interacting effects of orientation, material type, and TC on the tested properties were analyzed by using three-way ANOVA. All *p*-values less than 0.05 were considered statistically significant.

## 3. Results

Table 2 lists the three-way ANOVA results showing the interaction of all factors on trueness. The TC with the three different material types (ASIGA, NextDent, and FormLabs) showed no significant difference. The interaction between TC and orientation at 0°, 45°, and 90° showed no statistically significant difference, while different printing orientations at 0°, 45°, and 90° with different material types (ASIGA, NextDent and FormLabs) showed a statistically significant difference (*p* < 0.001). Also, no statistically significant difference was observed between all factors.

**Figure 4:**
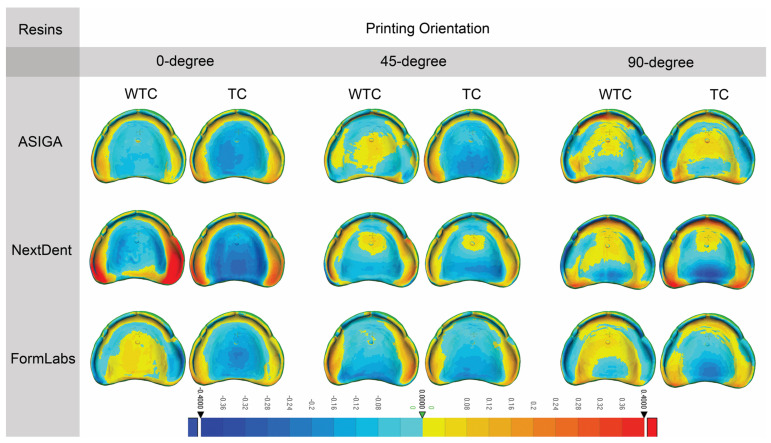
Representative sample from each group for trueness. “TC, Thermal cycling; WTC, Without thermal cycling”. The color bar indicates the magnitude of deviation in millimeters.

Table 3 shows the mean and standard deviation of trueness of the 3D-printed denture base resins in terms of orientation and TC effects. Figure 4 presents representative deviation maps for each printing orientation and resin type under TC and WTC conditions. Before TC, there was no statistically significant difference between 0° and 45 ° printing orientations, but it was significant in comparison with 90° printing orientation for ASIGA. In contrast, after TC, ASIGA was not statistically significant at all printing orientations. In addition, the effect of TC for ASIGA was significant at 0° printing orientation. Before and after TC, there was no statistically significant difference between 45° and 90° printing orientations, but it was significant in comparison with 0° printing orientation for NextDent. In addition, the effect of TC for NextDent was significant at 0° and 90° printing orientations. Before and after TC, there was no statistically significant difference between all printing orientations for FormLabs. The same result for the effect of TC for FormLabs was not significant across all printing orientations.

Table 4 lists the three-way ANOVA results showing the interaction of all factors on precision. The effect of different printing orientations and material types showed a statistically significant difference with a *p*-value < 0.01. No significant differences were observed between TC effect and material type, as well as between TC effect and printing orientation. In addition, no significant interaction was observed between the three factors.

**Figure 5:**
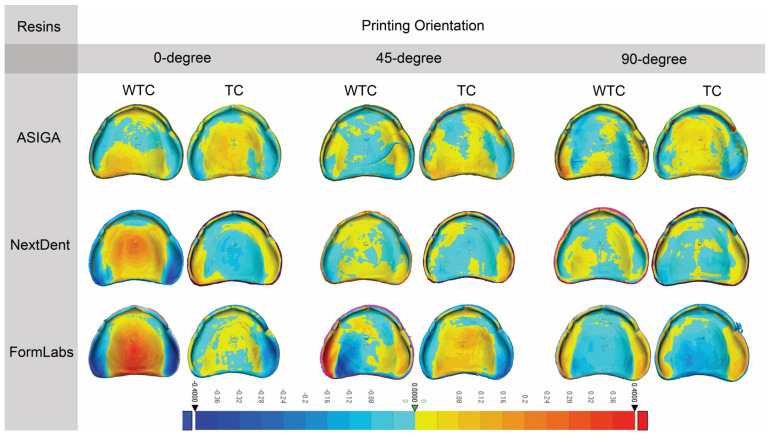
Representative sample from each group for precision. “TC, Thermal cycling; WTC, Without thermal cycling”. The color bar indicates the magnitude of deviation in millimeters.

Table 5 shows the mean and standard deviation of the precision of 3D-printed denture base resins in terms of orientation and TC. Figure 5 provides visual deviation profiles for the evaluated resins at various printing orientations in both TC and WTC groups. Before TC, no statistically significant difference was observed between 0° and 45° printing orientation, but it was significant in comparison with 90° printing orientation for ASIGA. After TC, ASIGA was not statistically significant at all printing orientations. Before TC, no statistically significant difference was shown between 45° and 90° in comparison with 0° for NextDent. After TC, no statistically significant effect was observed at all printing orientations for NextDent. Before TC, no statistically significant difference was exhibited at all printing orientations for FormLabs, while after TC, statistically significant effects were observed between all printing orientations for FormLabs. Overall, before and after TC, printing orientations at 0° and 90° were shown to be statistically significant for NextDent.

## 4. Discussion

This study investigated how different printing orientations affect the trueness and precision of 3D-printed denture bases fabricated with two different printing technologies: SLA and DLP. The null hypothesis posited that the three different printing orientations and material types would not result in significant differences in accuracy (trueness and precision). However, the research findings demonstrated significant variations across the three printing orientations and material type, thereby rejecting the null hypothesis.

The staircase effect and geometric shape of the denture can also affect accuracy [35,36]. Therefore, the object should be printed at an appropriate angle to control these factors and minimize errors. Regarding the improved clinical performance of dentures printed at 45°, the surface area of an object will be reduced by each layer’s thickness when printing at an angle; this decreases object removal from the resin tank and may enhance the success of the printing. In addition, denser support structures were observed at 45° in comparison to the other two printing orientations, creating more positive deviation in the area with more support. This finding is consistent with the results of previous studies [36,37].

The most unfavorable trueness value was observed at 0° printing orientation, indicating that the denture fit is less clinically favorable. This confirms the study by Tamaki et al. (2020), in which they used the same printing orientations, showing the highest trueness value at 0° printing orientation [36]. In addition, similar results were reported in a study by Choi et al. (2019) [38]. While the most favorable trueness value was shown at 45°, except for FormLabs, significant differences were observed between the printing orientations, which may be correlated to the type of resin and other factors. The results obtained in this study also align with those of Tamaki et al. (2020), in which the most favorable trueness value was observed at 45° [36]. Therefore, the best clinical fit for denture bases is 45° printing orientation.

In alignment with a recent systematic review conducted by AlGhamdi et al. [7], it was observed that the highest accuracy of 3D-printed denture bases was observed at a printing orientation of 45°, followed by 90°. The incorporation of additional struts and bars on the cameo surface further enhanced the accuracy of the 3D printing process. These findings underscore the significant impact of printing orientation on the accuracy of 3D-printed resin, with the 45° angle yielding the highest precision results. Additionally, factors such as the density and positioning of the support structures also play a critical role in influencing overall accuracy [7]. The highest precision value was observed at 0°, except at 45° for FormLabs, while the lowest value was shown for dentures printed at 45°, except for FormLabs. These results are similar to those reported for trueness. This result could be caused by printing near the center, resulting in higher accuracy and avoiding the platforms for which accuracy is poor [25].

The most unfavorable trueness value was shown for NextDent at 0°, which indicates that the denture fit is less clinically favorable. This result is similar to that of Alalawi et al. (2023) [32], in which they tested the accuracy of 3D-printed artificial teeth using the same resin materials used in this study. Their results indicated that NextDent had the most unfavorable trueness value at the axial surface, while FormLabs had the most unfavorable trueness value at the occlusal and cervical surfaces. Another study tested printing accuracy and flexural strength of the same 3D-printed resin materials used in this study, but at 90° printing orientation, finding that different resin materials had a significant influence on the printing accuracy [29]. However, their experiments involved bar specimens rather than denture models and used different printing technologies, which may contribute to the difference in results. In this study, the most favorable trueness value was shown for the ASIGA group at 0° and 45° with no significant difference, which indicates a more favorable clinical fit of the denture. This supports the results of a previous study in which ASIGA bar specimens exhibited the lowest error in all dimensions in comparison to NextDent and FormLabs [29].

The results of this study can be explained by the different resin materials, which are attributed to different chemical compositions at different printing orientations. In addition, a study by Segbaya et al. (2020) [39] indicated that printing materials with the same printer can produce different compositions due to the chemical characterization of 3D-printed resins. The precision value was the highest at 45° for FormLabs. While the lowest precision was at 45° and 90° for NextDent, with no significant difference between the values. These variable results can be explained by the type of resin materials, which can also influence the accuracy of denture fit. However, it is unclear whether the materials or the printing orientation had a more significant impact.

It is important to test dental materials after aging, as some materials with excellent characteristics at baseline testing may show severe deterioration following artificial aging. The most unfavorable trueness value after TC was at 0°, which indicates the least favorable clinical fit of the denture, while the lowest value was at 45°. These results are accompanied by the printing orientation factor when tested alone, which indicates that printing orientation is more influential than the TC factors. The highest precision value after TC was observed at 90°, and the lowest value was observed at 0°, indicating that TC has a stronger effect on precision than printing orientation. In contrast to Mudhaffer et al. [40], who tested flexural strength and modulus of elasticity at 0°, 45°, and 90° printing orientations after thermal aging (TA) using different resin materials, their results showed that, despite a higher filler load, NextDent had the lowest value of flexural strength after 3 months of TA. In addition, they confirmed that TA has a greater effect when it is prolonged, and its effect is material-specific.

The most unfavorable trueness value after TC was observed for NextDent at 0°, indicating a less favorable clinical fit of the denture. In addition, before TC, NextDent showed the most unfavorable trueness value, and the correlation between WTC and TC showed a significant difference, while the most favorable trueness value was observed for FormLabs at 45°. This result can be explained by the chemical composition of resin with TA, resulting in increased surface hardness; this affects the physical properties of the denture, thereby affecting the accuracy [41]. Effect of aging on dimensional accuracy and color stability of CAD-CAM milled and 3D-printed denture base resins: a comparative in vitro study. The highest precision value after TC was observed for FormLabs at 90°, followed by ASIGA at 90°, with the lowest value for FormLabs at 0°. TC can affect the accuracy of the 3D-printed denture by water sorption, which may change the physical properties of the 3D-printed resin [42]. Furthermore, the excessive number of residual monomers that leak after printing, incomplete bonding, and low polymerization rate can affect dimensional accuracy with TC [41].

As illustrated in the systematic review of Shahidi et al. (2020) [43], most printing technologies were shown to have acceptable clinical accuracy. The smallest mean error for SLA printers was 3.3 μm. In the present study, SLA and DLP technologies showed that each resin requires its own printing technology. ASIGA and Nextdent were printed with DLP, while FormLabs was printed with SLA. In the present study, the trueness and precision did not have a clear alignment based on the printing technology effect. This finding is in agreement with a previous study [11], which showed a variation between the two technologies in terms of precision and trueness, but in disagreement with a previous study [12] that compared DLP vs. SLA and found that SLA has superior accuracy. The difference in results may be attributed to the printers, materials, and printing parameters used. This finding indicates that the combination of different parameters results in significant differences. Previous studies investigated only one parameter effect, so further research on more parameter combinations is recommended [7].

The findings of this study indicate that multiple factors are correlated (printing orientation, the types of 3D-printed resins, and TC) and affect the accuracy of denture bases. The results show that the 45° printing orientation consistently demonstrated the highest accuracy, followed by 90°, with 0° yielding the poorest clinical fit of denture bases. This accuracy helps keep the denture balanced when occlusal force is applied. In addition, these printing orientations resulted in a favorable denture base fit at the posterior palate, which offers them better alignment with the anatomical structure of the edentulous maxilla [44,45]. The influence of resin types had varying effects, as ASIGA exhibited the most favorable trueness value, while NextDent showed the lowest value in precision. In addition, TC influenced precision, yet the interaction between TC and printing orientation did not have a significant effect on trueness. In contrast, TC had a complex effect on resin types, exhibiting the most favorable trueness value for FormLabs at 45° and the lowest precision for FormLabs at 0°. Although TC exhibited variable results for different types of resin, all the results in this study were within an acceptable clinical range. As was suggested, clinically acceptable deviation error should be less than 200 µm [46], while Hiroqaki et al. (2001) [47] suggested the error deviation should be less than 300 µm. In the present study, all values were within the clinically acceptable values. However, the results highlight that factors such as printing type, printing orientation, and aging may affect the trueness and precision of denture base resin. Ensuring that dimensions remain within the clinically acceptable range can help prevent issues such as poor fit, discomfort, or compromised functionality. Therefore, it is crucial for practitioners to take these findings into account when planning the fabrication of removable dentures, as some printers achieved better trueness and precision at specific orientations. For instance, ASIGA and FormLabs achieved better trueness at 0-degree orientation, while NextDent achieved better trueness when printed at 45-degree orientation. As a result, optimizing the best parameters for each printer is important to ensure the best clinical outcomes are achieved.

In terms of printing layer thickness, it should be considered in future investigations as a variable. It was reported that changing layer thickness affects the accuracy of printed specimens [44]. Due to the closed access of some printers, such as NextDent, the LT could not be considered in the present study. Therefore, further investigations with different layer thicknesses on the accuracy are recommended. The limitations of this study also include its in vitro nature, as the thermocycling method is not an actual replication of materials aging inside the oral cavity. In addition, while the study utilized common resin materials, more comprehensive analyses over extended cycling periods that incorporate additional resin types, post-curing conditions, and print geometries would provide deeper insights into the complex factors affecting denture accuracy. Clinical evaluations focusing on patient satisfaction would also be valuable in translating technical accuracy into actual masticatory performance. Although this study employed an SLA and DLP 3D printer, which are commonly used across various fields, the parameters were fixed and could not be adjusted freely. As a result, the same trends were not consistently observed when using other types of 3D printers. Further investigations are needed to study the mechanical properties of 3D-printed dentures, utilizing the same factors from this study, while also exploring different printing technologies and parameters. Future investigations should evaluate color stability, surface roughness, and the bonding performance of the printed denture base materials to denture teeth.

## 5. Conclusions

The findings of this study indicate that a 45° printing orientation yields the most accurate clinical fit, highlighting its significance in optimizing printing outcomes. Notably, ASIGA demonstrated the most favorable trueness value, while FormLabs exhibited the lowest precision, suggesting variability in performance among different systems. Furthermore, the influence of printing orientation on trueness was found to be more pronounced than the effects of TC. After undergoing TC, ASIGA displayed some significant variation in trueness, while NextDent showed some significant variations in both trueness and precision, indicating that aging may negatively affect the trueness and precision of these two printers despite the baseline performance.

## Figures and Tables

**Table 1:** Material used, specifications and specimens printing procedures.

Specifications, Printers, and Printing Parameters	3D-Printed Resins		
	ASIGA	NextDent	FormLabs
Brand name	DentaBASE (ASIGA, Erfurt, Germany)	Denture 3D+ (NextDent B.V., Soesterberg, The Netherlands)	Denture Base Resin LP (Formlabs Inc., Somerville, MA, USA)
Composition	Ethoxylated bisphenol A dimethacrylate 7,7,9 (or 7,9,9)-trimethyl-4,13-dioxo-3,14dioxa-5,12-diazahexadecane-1,16-diyl bismethacrylate 2-hydroxyethylmethacrylate, Silicon dioxide Diphenyl (2,4,6-trimethylbenzoyl)-phosphine oxide Titanium dioxide	Ester-based monomer Bisacylphosphine oxide (BAPO) Phenyl bis (2,4,6-trimethylbenzoyl)-phosphine oxide	55–75% *w*/*w* urethane dimethacrylate 15–25% *w*/*w* methacrylate monomers <0.9% *w*/*w* phenyl bis (2,4,6-trimethylbenzoyl)-phosphine oxide
Printer	ASIGA MAX™	NextDent 5100	Form 2
Printing technology	LED-based digital light processing (DLP)	Figure 2 DLP	Stereolithography (SLA)
Printing orientation	0° 45° 90°	0° 45° 90°	0° 45° 90°
Printing layer thickness	50 μm	50 μm	50 μm
Specimens cleaning after printing	Isopropyl Alcohol 99.9%	Isopropyl Alcohol 99.9%	Isopropyl Alcohol 99.9%
Post-curing conditions	Machine: Asiga-Flash Time: 20 min Temperature: 60 °C Wavelength: 405 nm	Machine: LC-D Print Box Time: 30 min Temperature: 60 °C Wavelength: 405 nm	Machine: FormCure Time: 30 min Temperature: 60 °C Wavelength: 395 nm
Support structure removal	Diamond discs	Diamond discs	Diamond discs
Finishing and Polishing	Silicon carbide grinding papers (500-then 1200-grit)	Silicon carbide grinding papers (500-then 1200-grit)	Silicon carbide grinding papers (500-then 1200-grit)
Storage	37 °C distilled water	37 °C distilled water	37 °C distilled water

**Table 2:** Three-Way ANOVA for the interacting effects of the factors on the trueness (RMS-mm).

	Sum of Squares	DF	Mean Square	F	*p*
Intercept	1.682	1	1.682	353.997	<0.001 *
TC effect * material type	0.009	2	0.004	0.938	0.394
TC effect * orientation	0.008	2	0.004	0.862	0.425
Orientation * material type	0.113	4	0.028	5.943	<0.001 *
TC effect * orientation * material type	0.036	4	0.009	1.919	0.112
Error	0.570	120	0.005		
Total	2.533	138			

* *p* < 0.05 statistically significant. TC, Thermal cycling.

**Table 3:** Mean, SD and significance of trueness (RMS-mm) of 3D-printed denture base resins in terms of orientation and thermal cycling effects.

Resins	TC	Printing Orientation			
		0-Degree	45-Degree	90-Degree	*p*
ASIGA	WTC	0.065(0.01) a	0.087(0.01) a	0.110(0.03)	<0.001 *
	TC	0.088(0.01) a	0.101(0.02) a	0.105(0.03) a	0.253
	P	0.001 *	0.102	0.694	
NextDent	WTC	0.240(0.02)	0.110(0.09) a	0.105(0.04) a	<0.001 *
	TC	0.170(0.06)	0.090(0.02) a	0.140(0.01) a	<0.001 *
	P	0.008 *	0.608	0.018 *	
FormLabs	WTC	0.069(0.05) a	0.166(0.26) a	0.093(0.06) a	0.509
	TC	0.085(0.01) a	0.077(0.02) a	0.083(0.02) a	0.717
	P	0.455	0.390	0.676	

*p < 0.05 statistically significant per material. Same small letters in each row show insignificant differences between the pairs. TC, Thermal cycling; WTC, Without thermal cycling.

**Table 4:** Three-Way ANOVA for the interacting effects of the factors of precision.

	Sum of Squares	DF	Mean Square	F	*p*
Intercept	0.677	1	0.677	177.166	<0.001 *
TC effect * material type	0.017	2	0.008	2.201	0.116
TC effect * orientation	0.007	2	0.003	0.911	0.405
Orientation * material type	0.057	4	0.014	3.756	0.007 *
TC effect * orientation * material type	0.028	4	0.007	1.807	0.133
Error	0.390	102	0.004		
Total	1.193	120			

* *p* < 0.05 statistically significant. TC, Thermal cycling.

**Table 5:** Mean, SD and significance of Precision (RMS-mm) of 3D-printed denture base resins in terms of orientation and thermal cycling effects.

Resins	TC	Printing Orientation			
		0-Degree	45-Degree	90-Degree	*p*
ASIGA	WTC	0.049(0.01) a	0.058(0.01) a	0.089(0.03)	0.006 *
	TC	0.059(0.02)	0.080(0.03)	0.083(0.02)	0.222
	P	0.331	0.110	0.719	
NextDent	WTC	0.135(0.04)	0.037(0.01) a	0.036(0.01) a	<0.001 *
	TC	0.061(0.05)	0.056(0.03)	0.047(0.01)	0.715
	P	0.011 *	0.12	0.036 *	
FormLabs	WTC	0.087(0.07)	0.178(0.23)	0.089(0.09)	0.489
	TC	0.042(0.01)	0.079(0.02)	0.086(0.03)	0.017 *
	P	0.170	0.311	0.937	

* *p* < 0.05 statistically significant per material. Same small letters in each row show insignificant differences between the pairs. TC, Thermal cycling; WTC, Without thermal cycling.

## Data Availability

The data supporting this study’s findings are available from the corresponding author upon reasonable request.
